# Development of a Peripheral Venous Catheter-Associated Phlebitis Risk Scale: A Methodological Study

**DOI:** 10.3390/jcm15114382

**Published:** 2026-06-05

**Authors:** Soner Berşe, Nuran Tosun, Betül Tosun

**Affiliations:** 1 Department of Nursing, Faculty of Health Sciences, Gaziantep University, Gaziantep 27310, Türkiye; 2 Department of Nursing, Faculty of Health Sciences, Hasan Kalyoncu University, Gaziantep 27410, Türkiye; nuran.tosun@hku.edu.tr; 3 Faculty of Nursing, Hacettepe University, Ankara 06230, Türkiye; betul.tosun@hacettepe.edu.tr

**Keywords:** peripheral venous catheter, phlebitis, risk assessment, scale development, validity, reliability, nursing care

## Abstract

**Background/Objectives:** To develop and validate a multidimensional risk assessment scale for identifying patients at risk of peripheral venous catheter (PVC)-associated phlebitis. **Methods:** This methodological study followed a two-phase design. In Phase 1 (scale development), an initial item pool of 39 candidate items was generated from a focused literature review and refined using the Lawshe technique with 20 expert raters. Data were collected from 729 hospitalized patients, who contributed 1008 PVCs between February and September 2021. Because the scale items are catheter-level, the PVC was the unit of analysis: 502 PVCs (from 380 patients) were used for exploratory factor analysis (EFA), and 506 PVCs (from 349 patients) for confirmatory factor analysis (CFA). In Phase 2 (clinical application), the finalized scale was administered to a separate, independent cohort of 208 patients between September and October 2021 alongside the Infusion Nurses Society (INS) Phlebitis Scale. Reliability was assessed using the Kuder–Richardson 20 (KR-20) coefficient, and discriminative performance was evaluated with Receiver Operating Characteristic (ROC) curve analysis. **Results:** EFA and CFA confirmed a three-factor structure comprising 14 items distributed across Individual, Chemical, and Mechanical risk domains. The instrument demonstrated strong internal consistency (KR-20 = 0.823) and excellent discriminative accuracy (AUC = 0.898), with an optimal cut-off of 20.5 (sensitivity 87%, specificity 91%). All CFA fit indices met the conventional acceptability thresholds (χ^2^/df = 3.249; GFI = 0.943; AGFI = 0.914; CFI = 0.942; NFI = 0.919; IFI = 0.943; TLI = 0.925; RMSEA = 0.067). In Phase 2, scale scores correlated significantly with the INS Phlebitis Scale (r = 0.794, *p* < 0.001). **Conclusions:** The Risk Assessment Scale for PVC-Associated Phlebitis is a valid and reliable instrument with strong psychometric properties. It enables early identification of high-risk patients and supports targeted preventive strategies in clinical practice.

## 1. Introduction

Peripheral venous catheters (PVCs) are among the most frequently used invasive devices in hospitalized patients, with approximately 80% of inpatients requiring at least one PVC during their stay for fluid therapy, medication administration, blood transfusion, parenteral nutrition, or hemodynamic monitoring [1,2,3,4,5,6]. Although PVCs provide rapid and cost-effective vascular access, they are associated with complications including infiltration, extravasation, and phlebitis. PVC-associated phlebitis manifests locally as pain, erythema, edema, tenderness, and palpable induration at the insertion site, but its clinical consequences extend well beyond local discomfort: it can lead to treatment interruption, dose loss, premature catheter removal, prolonged hospital stay, increased costs, loss of future venous access, and—if bacterial in origin and left untreated—progression to bloodstream infection, ultimately contributing to morbidity and mortality [7,8,9,10].

Reported incidence rates of PVC-associated phlebitis vary widely, from 0.1% to 63.3%, reflecting heterogeneity in patient populations, catheter-related practices, definitions of phlebitis, and assessment methods [11,12,13,14,15]. The Infusion Nurses Society (INS) recommends that phlebitis incidence be maintained below 5% across patient populations and that patients with PVCs be regularly monitored using reliable assessment tools [13,16]. Multiple patient-, treatment-, and device-related risk factors have been identified, including age, chronic illness, infused medications, catheter site, and catheter type [3,16,17].

However, most existing instruments focus on identifying the presence or severity of phlebitis after clinical signs have emerged, rather than systematically quantifying the cumulative contribution of patient- and catheter-related risk factors before phlebitis develops [8,13]. To the best of our knowledge, no validated multidimensional scale has been developed specifically to assess the risk of PVC-associated phlebitis. This conclusion was based on a targeted literature search conducted in PubMed, Scopus, Web of Science, CINAHL, and Google Scholar up to May 2026, using combinations of the terms “peripheral venous catheter,” “peripheral intravenous catheter,” “phlebitis,” “PVC-associated phlebitis,” “risk assessment,” “risk scale,” “prediction tool,” “scale development,” and “validation.” The search indicated that existing studies primarily focused on phlebitis incidence, risk factors, prevention, or post-event phlebitis assessment, whereas no methodological study specifically developing and validating a multidimensional PVC-associated phlebitis risk assessment scale was identified. A standardized risk-stratification tool would enable nurses to identify high-risk patients at the bedside and initiate targeted preventive measures in a timely manner. Accordingly, this methodological study aimed to develop and psychometrically validate a multidimensional Risk Assessment Scale for Peripheral Venous Catheter-Associated Phlebitis in hospitalized adult patients.

## 2. Methods

### 2.1. Study Design

This study employed a methodological design and was conducted in two phases. Phase 1 (scale development) comprised item generation, content validity assessment, and psychometric testing of the scale through exploratory and confirmatory factor analyses in a sample of 729 hospitalized patients (1008 PVCs). Phase 2 (clinical application) involved the administration of the finalized scale to a separate cohort of 208 patients to evaluate its real-world performance and concurrent validity against the Infusion Nurses Society (INS) Phlebitis Scale.

### 2.2. Study Population

Phase 1 was conducted in the Internal Medicine, General Surgery, Orthopedics and Traumatology, Cardiology, Infectious Diseases, and Oncology clinics of a Research and Training Hospital between February and September 2021. In scale development studies, a sample size of 5 to 10 times the number of scale items is recommended [18,19]; based on an initial draft of 22 items, a minimum of 220 participants was required. Data were collected from a total of 729 hospitalized patients, who contributed 1008 PVCs. For exploratory factor analysis (EFA), data from 502 PVCs (from 380 patients) were analyzed, while data from 506 PVCs (from 349 patients) were used for confirmatory factor analysis (CFA). A sample size of 1000 or more is considered excellent for factor analysis based on established standards for sample adequacy [18,20]; thus, the sample size of 1008 PVCs meets this criterion, underscoring the robustness of the dataset [20].

### 2.3. Inclusion and Exclusion Criteria

Patients hospitalized for at least three days at Gaziantep University Şahinbey Research and Training Hospital, aged 18 or older, with a PVC in place, and who voluntarily agreed to participate were included in the study. Patients hospitalized for less than three days or those who wished to withdraw from the study were excluded.

### 2.4. Data Collection Tools

Data were collected using the Patient Information Form, the INS Phlebitis Scale [21], and the Risk Assessment Scale for PVC-Associated Phlebitis.

### 2.5. Patient Information Form

This form, developed by the researcher based on a review of the relevant literature, includes sections for recording patient demographics and clinical details, such as age, height, weight, body mass index (BMI), sex, chronic diseases, dominant hand, diagnosis, hospitalization, and dates of admission and discharge.

### 2.6. INS Phlebitis Scale

The scale categorizes phlebitis into five grades as follows: Grade 0, no symptoms; Grade 1, redness and/or pain at the access site; Grade 2, pain at the access site with redness and/or edema; Grade 3, pain at the access site with redness and/or edema, streak formation, and palpable venous cord; and Grade 4, redness, pain and/or edema at the access site, streak formation, palpable venous cord, a palpable venous cord greater than 2.5 cm in length, and purulent discharge.

### 2.7. Development of the Risk Assessment Scale for Peripheral Venous Catheter-Associated Phlebitis

#### 2.7.1. Initial Item Pool Creation

An initial pool of 39 items was developed based on an exhaustive review of existing literature, aimed at capturing a wide range of potential risk factors for PVC-associated phlebitis.

#### 2.7.2. Expert Validation and Refinement

To establish content validity, the 39-item pool was reviewed by a panel of 20 experts, including university faculty and practicing clinical nurses. The experts assessed each item for necessity, relevance, and clarity using the Lawshe technique. Items were rated as “necessary,” “useful but needing modification,” or “unnecessary.” Based on input from experts, some items were refined for clarity and relevance, while items that did not meet the content validity ratio (CVR) threshold of 0.42 were removed. After expert review and refinement, the item pool was reduced to 22 items. The final version of the scale is provided in Appendix A.

### 2.8. Exploratory Factor Analysis (EFA)

The refined 22-item scale was subjected to exploratory factor analysis (EFA) to identify its underlying factor structure. EFA led to the removal of 8 additional items that did not significantly contribute to the scale’s reliability or validity. Redundant items or items with low factor loadings were excluded, resulting in a final scale of 14 items.

### 2.9. Confirmatory Factor Analysis (CFA)

To validate the factor structure from EFA, confirmatory factor analysis (CFA) was performed with a different sample group. The CFA model showed good fit indices, supporting a three-factor model as an accurate representation of the data. Fit indices included Adjusted Goodness-of-Fit Index (AGFI) = 0.914), Tucker–Lewis Index (TLI) = 0.925, and Goodness-of-Fit Index (GFI) = 0.943, all indicating a good model fit. The Root Mean Square Error of Approximation (RMSEA) was 0.067, and the χ^2^/df ratio was 3.24, further supporting the model’s adequacy.

Organization into Subscales: The final 14-item scale was organized into three subscales:

Individual Risk Factors: This subscale includes items related to patient-specific characteristics, such as age and the presence of chronic diseases, that may predispose patients to phlebitis.

Chemical Risk Factors: This subscale encompasses items related to the chemical properties of infused solutions and medications, including chemotherapy drugs and the osmolarity of administered fluids.

Mechanical Risk Factors: This subscale covers items related to the mechanical aspects of catheter use, such as catheter size, catheter dwell time, and the number of insertion attempts.

### 2.10. Scoring System

Each item on the scale is assigned a score of 2 points for “Yes” responses and 1 point for “No” responses. Higher scores indicate greater risk of PVC-associated phlebitis. The scale does not include any reverse-scored items, simplifying the scoring process.

The decision to score items as “Yes” = 2 and “No” = 1, rather than the conventional 1/0 binary, was made on clinical and conceptual grounds. Because every hospitalized patient with an in situ PVC inherently carries some baseline level of phlebitis risk by virtue of having an indwelling intravascular device, a scoring system in which the absence of a risk factor yields a value of zero was considered conceptually misleading, as it could imply a complete absence of risk. The 1/2 coding ensures that each assessed risk factor contributes a measurable weight to the total score while preserving the binary nature of the items. It should be noted that this scoring approach is mathematically equivalent to a 0/1 coding—the two systems differ only by a constant shift in the total score—and therefore does not alter the psychometric properties of the scale, including item-total correlations, KR-20 reliability, factor structure, or ROC-based discriminative performance.

Based on the ROC curve analysis, the optimal cut-point was set at 20.5, yielding a sensitivity of 87% and a specificity of 91%.

### 2.11. Internal Consistency and Reliability Testing

The internal consistency of the scale was assessed using the Kuder–Richardson 20 (KR-20) formula, which produced a reliability coefficient of 0.823, indicating high reliability.

### 2.12. Face Validity and Pilot Testing

To assess the clarity, length, and readability of the draft scale items, a face validity analysis was conducted. The 22-item draft scale was administered to a pilot sample of 50 participants selected based on the study’s inclusion and exclusion criteria. At this stage, each item was evaluated for clarity, comprehensibility, and feasibility. No items were identified as difficult to understand or requiring additional clarification.

### 2.13. Application of the Final Scale

Following the completion of Phase 1 (scale development), the finalized 14-item Risk Assessment Scale for PVC-Associated Phlebitis was administered in Phase 2 (clinical application) between September and October 2021 to a separate, independent cohort of 208 hospitalized patients—distinct from the 729 patients of Phase 1—recruited using the same inclusion and exclusion criteria from the same clinical wards (Cardiology, General Surgery, Oncology, Internal Medicine, Orthopedics and Traumatology). Each patient was assessed during the dwell time of their PVC over a four-day follow-up period (Days 1–4 after catheter insertion). The Infusion Nurses Society (INS) Phlebitis Scale [21] was administered concurrently to the same patients during the same observation period to assess the concurrent validity of the new instrument.

### 2.14. Statistical Analysis

Data analysis was performed using SPSS software for Windows, version 25.0 (IBM Corp., Armonk, NY, USA), in conjunction with IBM SPSS Amos, version 25.0, for structural equation modeling.

Descriptive statistics are reported as frequency, percentage, or mean and standard deviation, as appropriate. The normality of data distribution was checked using normality tests and by examining skewness and kurtosis metrics. The Kaiser-Meyer-Olkin (KMO) measure was utilized to verify the adequacy of the sample size before performing EFA. To evaluate the reliability and construct validity of the developed scale, both EFA and CFA were conducted. The reliability of the scale was assessed using the KR-20. Additionally, Receiver Operating Characteristic (ROC) curve analysis was employed to determine the optimal threshold value for the newly developed scale. Because the items of the scale describe characteristics of the individual PVC (e.g., catheter gauge, dwell time, infused medications, insertion attempts, three-way stopcock use) rather than only patient-level attributes, the PVC was treated as the unit of analysis. In the EFA sub-sample, 502 PVCs were obtained from 380 patients (PVC-to-patient ratio: 1.32); the majority of patients contributed a single catheter, while a smaller subset contributed two or more PVCs, mostly as a result of elective replacement after the recommended dwell time or clinically indicated removal. To assess the potential impact of within-patient clustering on the psychometric findings, a post hoc sensitivity analysis was performed in which one PVC per patient was randomly selected (*n* = 380) and the EFA was re-run; this procedure was repeated 10 times with different random seeds.

### 2.15. Ethical Considerations

Approval for the study was obtained from the Non-Interventional Research Ethics Committee of Hasan Kalyoncu University, Faculty of Health Sciences (Date: 19 January 2021, Decision No: 2021/003).

The subjects were informed that their participation was voluntary and that they have the right to withdraw at any time. Verbal and written consent were obtained from each participant.

## 3. Results

Of the participants included in EFA (*n* = 380), 69.7% were male, 31.3% had a chronic disease, and 20.8% were smokers. The mean age of the patients was 53.15 ± 18.95 years, and the mean BMI was 27.01 ± 5.40 kg/m^2^. Phlebitis was observed in 27.3% of the patients, with Grade 1 phlebitis occurring in 20.8% (Table 1).

Among the participants included in CFA (*n* = 349), 65.3% were male, 33.5% had a chronic disease, and 27.5% were smokers. The patients had a mean age of 55.21 ± 17.38 years and a mean BMI of 27.53 ± 5.65 kg/m^2^. In this group, the prevalence of phlebitis was 29.6%, with Grade 1 phlebitis present in 25.3% of the patients (Table 1).

### 3.1. Validity of the Risk Assessment Scale for PVC-Associated Phlebitis

The KMO value was 0.800, indicating adequate sampling suitability for factor analysis. Bartlett’s test of sphericity was significant, χ^2^(91) = 2552.827, *p* < 0.001, indicating that the correlation matrix was not an identity matrix and that the items were sufficiently intercorrelated for factor analysis. EFA was conducted using Principal Component Analysis with Varimax rotation to examine the underlying factor structure of the newly developed scale. Construct validity was further evaluated through both EFA and CFA (Table 2).

The construct validity of the 14-item scale was assessed through EFA. Factor loadings ranged from 0.804 to 0.825 for the first subscale (Individual Risk Factors), from 0.686 to 0.713 for the second subscale (Chemical Risk Factors), and from 0.543 to 0.782 for the third subscale (Mechanical Risk Factors). Items that overlapped and reduced the KR-20 value were excluded from the analysis. The scale items explained 58.09% of the total variance, confirming the three-factor structure (Table 3).

**Figure 1:**
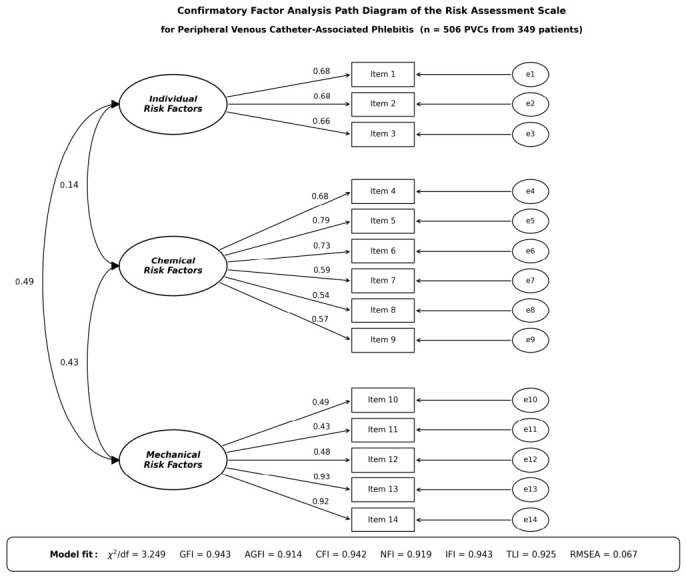
Confirmatory factor analysis (CFA) path diagram of the Risk Assessment Scale for Peripheral Venous Catheter-Associated Phlebitis (*n* = 506 PVCs from 349 patients). Items 1–14 correspond to the scale items listed in Table 3, and e1–e14 indicate the residual/error terms for the observed variables.

Analysis using structural equation modeling (SEM) revealed significant results (*p* ≤ 0.001), affirming the relevance of all items and the scale’s multifactorial structure. The model was subsequently refined for improved alignment. The goodness-of-fit indices for the first-order multifactor analysis of the newly developed scale indicated a good fit, with an AGFI of 0.914, a TLI of 0.925, and a GFI of 0.943, while the fit was considered acceptable based on a χ^2^ value of 3.249, a RMSEA of 0.067, along with Comparative Fit Index (CFI), Normed Fit Index (NFI), and Incremental Fit Index (IFI) values of 0.942, 0.919, and 0.943, respectively (Table 4). The standardized factor loadings and the three-factor structure of the scale are illustrated in Figure 1.

### 3.2. Reliability of the Risk Assessment Scale for PVC-Associated Phlebitis

To evaluate item discrimination and internal consistency, corrected item–total correlations, independent samples *t*-tests between the upper and lower 27% groups, and KR-20 reliability coefficients were examined. A corrected item–total correlation threshold of 0.40 was used to indicate satisfactory item discrimination. In the present study, corrected item–total correlations ranged from 0.514 to 0.645, showing that all items exceeded the predefined threshold and contributed adequately to the scale. The independent samples *t*-tests between the upper and lower 27% groups were statistically significant for all items, further supporting the discriminative power of the items (Table 5).

To assess the distinctiveness of the items on the scale, the scores were organized in descending order. Then, the average scores of the lowest and highest 27th percentile groups were analyzed using an independent samples *t*-test. This analysis revealed a statistically significant difference between the mean scores of the two groups, suggesting that the scale effectively distinguishes the attribute in question, consistent with prior reports [22].

Reliability analysis was conducted to determine whether the scale items were consistent with one another and whether all items measured the same construct [22]. Reliability analysis revealed a KR-20 coefficient of 0.823, indicating that it is a highly reliable scale. Furthermore, to assess the robustness of these psychometric findings against potential within-patient clustering (see Section 2.13), a post hoc sensitivity analysis was performed. In 10 random replications with one PVC per patient (*n* = 380), all key parameters remained essentially unchanged compared with the primary PVC-level analysis (KMO 0.781 ± 0.005; total explained variance 57.12% ± 0.28%; KR-20 0.809 ± 0.004), and the three-factor structure was preserved in 10/10 replications, with all 14 items consistently loading on their expected factor.

### 3.3. Scale Scoring

This instrument consists of 14 items, categorized under three distinct subscales: “Individual Risk Factors” (3 items), “Chemical Risk Factors” (6 items), and “Mechanical Risk Factors” (5 items). Utilizing a binary scoring system, the instrument assigns 2 points for “Yes” responses and 1 point for “No” responses. The scale has no reverse-scored items. The score ranges for the three subscales are as follows: 3 to 6 points for the Individual Risk Factors subscale, 6 to 12 points for the Chemical Risk Factors subscale, and 5 to 10 points for the Mechanical Risk Factors subscale. Possible total scores range from 14 to 28.

The area under the ROC curve for the scale was as 0.898. Through ROC analysis, the sensitivity and specificity of the scale were examined at different cut-points. The cut-off point of the scale (i.e., the point that best discriminates between positive and negative outcomes) was 20.5, with 87% sensitivity and 91% specificity (Table 6).

In the sensitivity analysis (10 random replications with one PVC per patient, *n* = 380), all key psychometric parameters remained essentially unchanged compared with the primary PVC-level analysis (KMO 0.781 ± 0.005; total explained variance 57.12% ± 0.28%; KR-20 0.809 ± 0.004), and the three-factor structure was preserved in 10/10 replications, with all 14 items consistently loading on their expected factor.

### 3.4. Results from Administration of the Final Scale

There was no significant difference in the risk of PVC-associated phlebitis between females and males. The following factors were significantly associated with an increased risk of phlebitis: patient age, presence of chronic illness, palpability of veins, continuous infusion, osmolarity of administered IV fluids ≥ 500 mOsm, IV drug administration, administration of IV push antibiotics, use of chemotherapeutic drugs, use of sedative medications, catheter dwell time of ≥72 h, catheter size < 20 G, history of catheter-related complications, multiple insertions of a catheter, and use of a three-way stopcock.

### 3.5. Correlation with the INS Phlebitis Scale

A significant and positive correlation was found between the scores for newly developed scale and the INS Phlebitis scale scores (r = 0.794, *p* < 0.001).

## 4. Discussion

This study presents the development and full psychometric validation of the Risk Assessment Scale for PVC-Associated Phlebitis (RAS-PVCP)—to our knowledge, the first multidimensional instrument designed specifically to quantify risk rather than grade severity of PVC-associated phlebitis. The instrument demonstrated strong content and construct validity, satisfactory internal consistency, and excellent discriminative performance.

### 4.1. Psychometric Properties

The suitability of the data for EFA was supported by a KMO value of 0.800 and a significant Bartlett’s test of sphericity, indicating that the correlation matrix was not an identity matrix and that the items were sufficiently intercorrelated for factor analysis. The three-factor solution—Individual, Chemical, and Mechanical Risk Factors—accounted for 58.09% of the variance, exceeding the 50% benchmark commonly considered adequate for multifactor instruments [22,23]. CFA in an independent subsample reproduced the structure, with all goodness-of-fit indices within acceptable to good thresholds (Table 4). Internal consistency was satisfactory (KR-20 = 0.823 overall; subscale range 0.774–0.827). Corrected item–total correlations ranged from 0.514 to 0.645, indicating that all items exceeded the predefined satisfactory threshold of 0.40 and contributed adequately to the scale.

### 4.2. Comparison with Existing Phlebitis Assessment Instruments

The scale should be interpreted in relation to existing phlebitis assessment instruments rather than as a replacement for them. The Visual Infusion Phlebitis (VIP) scale, the INS Phlebitis Scale [21], and the Maddox and Baxter phlebitis scales—summarized in previous reviews [8]—are primarily designed to standardize the recognition and grading of phlebitis after clinical signs such as pain, erythema, edema, induration, or venous cord have appeared. Similarly, the PIVC-miniQ [2] is valuable for monitoring the quality of peripheral intravenous catheter care. These instruments are essential for surveillance and clinical documentation; however, they do not combine individual, chemical, and mechanical risk factors into a single prospective risk score before or during catheter dwell.

This distinction is clinically important: although severity-grading tools are essential for documenting established phlebitis, they cannot identify high-risk patients in advance. Given that institutional phlebitis rates frequently exceed the <5% threshold recommended by international standards [13,16], a complementary risk assessment instrument may help close this preventive gap. The present scale therefore complements existing severity-grading and quality-monitoring tools by adding a structured method for identifying patients who may need preventive action before overt phlebitis develops. Direct psychometric comparison should be interpreted cautiously, because these tools serve different clinical purposes and report different indices; nevertheless, the present findings suggest that RAS-PVCP offers a clinically meaningful risk assessment function that is not provided by current grading tools.

### 4.3. Relevance to Clinical Practice and Future Directions

PVCs are among the most frequently used invasive devices in hospitalized patients, with up to 80% of inpatients requiring at least one PVC during their stay [1,2,3,4,5,6]. Even when not life-threatening, phlebitis can lead to pain, catheter removal, repeated cannulation, interruption of IV therapy, increased nursing workload, and additional healthcare costs [1,8,12,14]. The clinical value of the proposed scale lies in shifting practice from reactive assessment of established phlebitis to proactive risk recognition. The scale may be applied at catheter insertion and repeated during subsequent assessments, particularly when medications, infusion characteristics, dwell time, or catheter-related conditions change.

The multidimensional structure of the scale has direct implications for nursing decision-making. Individual risk factors may alert nurses to patients requiring closer site inspection, careful vein selection, patient education, and more frequent reassessment. Chemical risk factors—such as chemotherapeutic drugs, high-osmolarity infusates, IV drug administration, IV push antibiotics, and sedative medications—can prompt review of infusion rate, dilution, compatibility, route of administration, and the need for alternative vascular access when clinically indicated [13,16,17,24]. Mechanical risk factors—including catheter gauge, dwell time, multiple insertion attempts, history of catheter-related complications, and three-way stopcock use—can guide decisions about catheter selection, insertion technique, replacement planning, and avoidance of unnecessary manipulation of the catheter system [5,13,25,26].Thus, the scale not only produces a total score but also helps nurses identify which risk domain should be targeted in the care plan.

In routine practice, the new scale may be used alongside existing severity-grading tools, such as the INS Phlebitis Scale, rather than replacing them. A high risk score would indicate the need for preventive strategies and intensified monitoring, while the INS or VIP scale would continue to support documentation and management once clinical signs are present [8,13,17]. Because the items are largely based on routinely assessed clinical and catheter-related variables, the scale may be feasible for integration into electronic health records, vascular access checklists, nursing handbooks, and IV-therapy education programs [1,2,13,16]. Its use may be particularly relevant in oncology, critical care, infectious diseases, and surgical wards, where irritant medications, prolonged therapy, difficult venous access, and treatment interruptions can make PVC complications especially burdensome [4,17,24,26]. Future studies should test whether scale-guided preventive protocols reduce phlebitis incidence, re-cannulation, treatment interruptions, and catheter-related costs in prospective, multicentre, or interventional designs.

### 4.4. Limitations

Several limitations should be acknowledged. First, the study was conducted in a single tertiary research hospital, which may limit the generalizability of the findings. Second, the cross-sectional design does not allow for assessment of the scale’s predictive validity over time; longitudinal validation studies are warranted. Third, patients hospitalized for less than three days were excluded, potentially omitting cases of very early-onset phlebitis. Fourth, although the PVC was the appropriate unit of analysis given that the scale items are catheter-level, within-patient correlation arising from repeated PVCs in the same individual was not formally modeled in the primary analyses. The PVC-to-patient ratio was 1.32, indicating that the majority of patients contributed a single PVC. A post hoc sensitivity analysis restricting the analysis to one randomly selected PVC per patient confirmed the robustness of the three-factor structure and yielded essentially unchanged KMO, KR-20, and explained-variance values. Nevertheless, future validation studies should employ generalized estimating equations (GEE) or generalized linear mixed models with a patient-level random intercept to formally account for this clustering. Finally, the scale was developed and validated in a Turkish-speaking population; cross-cultural translation and validation in other languages and healthcare systems are recommended.

### 4.5. Suggestions

It is recommended that the new scale be incorporated into routine nursing practice for patients with PVCs, used alongside severity-grading instruments such as the INS Phlebitis Scale, and further validated across different patient populations and healthcare settings.

## 5. Conclusions

The Risk Assessment Scale for Peripheral Venous Catheter-Associated Phlebitis (RAS-PVCP) is a valid and reliable instrument for identifying the risk of PVC-associated phlebitis. Its 14 items show strong coherence and good internal consistency; both EFA and CFA support the three-factor structure in independent sub-samples; and its discriminative accuracy is high (AUC = 0.898; sensitivity 87%, specificity 91% at a cut-off of 20.5). To improve clinical outcomes, we recommend integrating this scale into routine nursing workflows and electronic health records for early risk identification. Furthermore, the scale should be used as a clinical teaching tool and validated in diverse healthcare settings through longitudinal and randomized studies to confirm its global applicability.

### Relevance to Clinical Practice

The newly developed Risk Assessment Scale for Peripheral Venous Catheter-Associated Phlebitis holds practical utility for nurses caring for patients with peripheral venous catheters and can serve as a valuable educational tool in nursing training programs. Moreover, the scale has the potential to contribute to experimental research focused on risk reduction. Specifically, it is recommended for identifying patients at risk for phlebitis and for planning, implementing, and evaluating their care. Additionally, translating the scale into various languages and conducting validity and reliability studies in diverse patient populations would be beneficial.

The lack of a national and local phlebitis risk assessment scale underscores the importance of this study. Integrating these findings into clinical practice is expected to improve phlebitis prevention and provide a more comprehensive assessment of phlebitis risk.

## Figures and Tables

**Table 1:** Descriptive Characteristics of Patients.

Descriptive Characteristics	EFA (*n* = 380 Patients)	CFA (*n* = 349 Patients)
**Sex, *n* (%)**		
Male	265 (69.7)	228 (65.3)
Female	115 (30.3)	121 (34.7)
**Age (years)**		
18–40, *n* (%)	112 (29.5)	91 (26.1)
41–64, *n* (%)	140 (36.8)	135 (38.7)
≥65, *n* (%)	128 (33.7)	123 (35.2)
Mean ± SD	53.15 ± 18.95	55.21 ± 17.38
Min–Max	18–94	18–90
**Body Mass Index (kg/m^2^)**		
<18.5 (Underweight), *n* (%)	14 (3.7)	10 (2.9)
18.5–24.9 (Normal), *n* (%)	135 (35.5)	113 (32.4)
≥25.0 (Overweight/Obese), *n* (%)	231 (60.8)	226 (64.8)
Mean ± SD	27.01 ± 5.40	27.53 ± 5.65
Min–Max	15.21–51.11	15.62–54.97
**Chronic Disease, *n* (%)**		
Yes	119 (31.3)	117 (33.5)
No	261 (68.7)	232 (66.5)
**Smoking Status, *n* (%)**		
Yes	79 (20.8)	96 (27.5)
No	301 (79.2)	253 (72.5)
**Dominant Hand, *n* (%)**		
Right	340 (89.5)	314 (90.0)
Left	40 (10.5)	35 (10.0)
**Clinical Ward, *n* (%)**		
Orthopedics and Traumatology	94 (24.7)	46 (13.2)
General Surgery	88 (23.2)	115 (33.0)
Oncology	87 (22.9)	27 (7.7)
Internal Medicine	65 (17.1)	51 (15.6)
Cardiology	46 (12.1)	80 (22.9)
Infectious Diseases	0 (0.0)	30 (8.6)
**Total**	**380 (100)**	**349 (100)**

EFA: Exploratory Factor Analysis; CFA: Confirmatory Factor Analysis; SD: Standard Deviation; Max: Maximum. The total study sample comprised 729 unique patients and 1008 PVC observations.

**Table 2:** Factor Structure and Explained Variance Ratio of the Risk Assessment Scale for PVC-Associated Phlebitis.

Factors	Variance (%)	Eigenvalue (Λ)
1. Individual Risk Factors	10.981	1.537
2. Chemical Risk Factors	31.533	4.415
3. Mechanical Risk Factors	15.576	2.181
Total Explained Variance	58.090	

**Table 3:** Item Factor Loadings of the Risk Assessment Scale for Peripheral Venous Catheter-Associated Phlebitis in EFA.

Scale Items	Individual Risk Factors	Chemical Risk Factors	Mechanical Risk Factors
Item 1. Difficulty palpating veins	0.825		
Item 2. Age 65 years or older	0.808		
Item 3. Presence of a chronic disease	0.804		
Item 4. Use of chemotherapeutic drugs		0.768	
Item 5. Peripheral administration of fluids with an osmolarity > 500 mOsm		0.767	
Item 6. Continuous infusion		0.713	
Item 7. IV drug administration		0.708	
Item 8. Administration of sedative medication		0.689	
Item 9. Peripheral administration of IV push antibiotics		0.686	
Item 10. Use of a three-way stopcock			0.782
Item 11. Catheter size < 20 G			0.756
Item 12. Catheter dwell time ≥ 72 h			0.743
Item 13. Two or more attempts to insert the catheter			0.557
Item 14. History of catheter-related complications			0.543

**Table 4:** Distribution of CFA Fit Index Values for the Risk Assessment Scale for Peripheral Venous Catheter-Associated Phlebitis (*n* = 506).

Goodness-of-Fit Measures	Criteria for Perfect Fit	Criteria for Acceptable Fit	Application Results	Result
CMIN/Df	0 ≤ χ^2^/df ≤ 3	3 ≤ χ^2^/df ≤ 5	3.249	Good fit
GFI	0.90 ≤ GFI	0.80 ≤ GFI	0.943	Perfect Fit
AGFI	0.90 ≤ AGFI	0.80 ≤ AGFI	0.914	Perfect Fit
CFI	0.95 ≤ CFI	0.85 ≤ CFI	0.942	Good fit
RMSEA	0.0 ≤ RMSEA ≤ 0.05	0.06 ≤ RMSEA ≤ 1.0	0.067	Good fit
NFI	0.95 ≤ NFI	0.80 ≤ NFI	0.919	Good fit
TLI	0.90 ≤ TLI	0.80 ≤ TLI	0.925	Perfect Fit
IFI	0.95 ≤ IFI	0.85 ≤ IFI	0.943	Good fit

**Table 5:** Item-Total Correlation Analysis.

Factors	Item	Item-Total Score Correlation	t (Bottom 27–Top 27%)	*p*-Value (Bottom 27–Top 27%)
**Chemical Risk Factors: KR20 = 0.827**				
Item 4		0.630	18.00	<0.001 ***
Item 5		0.588	23.345	<0.001 ***
Item 6		0.605	77.363	<0.001 ***
Item 7		0.572	43.646	<0.001 ***
Item 8		0.645	15.482	<0.001 ***
Item 9		0.561	26.449	<0.001 ***
**Mechanical Risk Factors: KR20 = 0.774**				
Item 10		0.530	25.100	<0.001 ***
Item 11		0.514	31.820	<0.001 ***
Item 12		0.558	25.100	<0.001 ***
Item 13		0.563	31.820	<0.001 ***
Item 14		0.569	31.820	<0.001 ***
**Individual Risk Factors: KR20 = 0.783**				
Item 1		0.631	46.476	<0.001 ***
Item 2		0.616	46.476	<0.001 ***
Item 3		0.615	33.000	<0.001 ***

Kuder–Richardson 20 (KR-20) = 0.823. The lower and upper 27% groups each included 136 observations. *** *p* < 0.001.

**Table 6:** ROC Analysis Results.

AUC *	Standard Deviation	*p*-Value	Cut-Point	Diagnostic Accuracy (%)	
				Sensitivity	Specificity
0.898	0.010	0.00	20.5	87%	91%

* AUC: Area Under the ROC Curve.

## Data Availability

The datasets generated and analyzed during the current study are not publicly available due to participant confidentiality restrictions but are available from the corresponding author on reasonable request.

## Appendix A

**Table A1:** Risk Assessment Scale for Peripheral Venous Catheter-Related Phlebitis (Draft Scale).

	Risk Factors	Yes	No
**Individual Risk Factors**	**(1)** Female sex		
	**(2)** Age 65 years or older		
	**(3)** Presence of a chronic illness (e.g., diabetes, cardiovascular disease, hypertension, peripheral artery disease)		
	**(4)** Smoking		
	**(5)** BMI ≥ 30 kg/m^2^ or BMI < 18.5 kg/m^2^		
	**(6)** Difficulty palpating veins		
**Chemical Risk Factors**	**(7)** Peripheral administration of fluids with an osmolarity > 500 mOsm (e.g., total parenteral nutrition, blood and blood products, Gelofusine)		
	**(8)** Continuous infusion		
	**(9)** IV drug administration (e.g., amiodarone, potassium, antibiotics)		
	**(10)** Peripheral administration of IV push antibiotics		
	**(11)** Use of chemotherapeutic drugs		
	**(12)** Administration of sedative medications (e.g., Midazolam, Propofol)		
**Mechanical Risk Factors**	**(13)** Catheter dwell time ≥ 72 hours		
	**(14)** Flow rate of IV infusion pump ≥ 90 ml/hour		
	**(15)** Placement of peripheral IV catheter in lower extremity		
	**(16)** Placement of catheter on wrist or back of the hand		
	**(17)** Catheter size < 20G		
	**(18)** Not using hypoallergenic and transparent fixation material		
	**(19)** History of catheter-related complications (e.g., infiltration, phlebitis, extravasation)		
	**(20)** Multiple insertions in the same vein where catheter is placed		
	**(21)** Two or more attempts to insert the catheter		
	**(22)** Use of a three-way stopcock		
